# The Potential of Spent Coffee Grounds @ MOFs Composite Catalyst in Efficient Activation of PMS to Remove the Tetracycline Hydrochloride from an Aqueous Solution

**DOI:** 10.3390/toxics11020088

**Published:** 2023-01-17

**Authors:** Wei Zhang, Jiajia Lu, Shoushu Liu, Chen Wang, Qiting Zuo, Lin Gong

**Affiliations:** 1 School of Ecology and Environment, Zhengzhou University, 100 Kexue Avenue, Zhengzhou 450001, China; 2 Yellow River Institute for Ecological Protection and Regional Coordination Development, Zhengzhou University, 100 Kexue Avenue, Zhengzhou 450001, China; 3 Henan Key Laboratory of Water Pollution Control and Rehabilitation Technology, Pingdingshan 467036, China; 4 Henan International Joint Laboratory of Water Cycle Simulation and Environmental Protection, Zhengzhou 450001, China; 5 Zhengzhou Key Laboratory of Water Resource and Environment, Zhengzhou 450001, China; 6 School of Water Conservancy Engineering, Zhengzhou University, Zhengzhou 450001, China

**Keywords:** tetracycline hydrochloride, CG@ZIF-67 composite, utilization of natural waste, peroxymonosulfate

## Abstract

The efficient removal of Tetracycline Hydrochloride (TC) from wastewater, which is a difficult process, has attracted increasing attention. Aiming to synchronously achieve the goal of natural waste utilization and PMS activation, we have combined the MOFs material with waste coffee grounds (CG). The catalytic activity of the CG@ZIF-67 composite in the TC removal process was thoroughly evaluated, demonstrating that the TC removal rate could reach 96.3% within 30 min at CG@ZIF-67 composite dosage of 100 mg/L, PMS concertation of 1.0 mM, unadjusted pH 6.2, and contact temperate of 293.15 K. The ^1^O_2_ and ·SO_4_^−^ in the CG@ZIF-67/PMS/TC system would play the crucial role in the TC degradation process, with ^1^O_2_ acting as the primary ROS. The oxygen-containing functional groups and graphite N on the surface of CG@ZIF-67 composite would play a major role in efficiently activating PMS and correspondingly degrading TC. In addition, the CG@ZIF-67/PMS/TC system could withstand a wide pH range (3–11). The application of CG in preparing MOF-based composites will provide a new method of removing emerging pollutants from an aqueous solution.

## 1. Introduction

In the water environment, the treatment of antibiotics pollutants, a typical kind of emerging pollutants [1,2], has received increasing attention [3,4,5,6,7]. Among the antibiotics pollutants, as the second most used antibiotic in the world [8], tetracycline hydrochloride (TC) has been widely used for many bacterial infections in humans and animals, since the majority of TC is hardly absorbed and up to 80–90% of the initial value or its metabolites can emit to the environment [9]. Though the concentration of TC in the environment is quite limited (from μg/L to ng/L) [10], higher TC concentrations (100–500 mg/L) were detected in hospital and pharmaceutical plant wastewater [11]. Due to its high persistence, biological activity, antibiotic resistance, and toxic effects on ecosystems [12], TC may lead to contamination of the environment, water resources, and soil. Many treatment methods to remove TC from an aqueous solution were investigated, e.g., adsorption [13], photocatalysis [14], electrocatalysis [15], and the Fenton oxidation processes [16]. Among those TC removal routes, the advanced oxidation processes (AOPs) based on persulfate (PS) have attracted increasing attention in recent years [17,18]; this is because the sulfate radical (·SO_4_^−^)-based AOPs have superior oxidation potential (E_0_ = 2.5–3.1 V), a wider pH operating range, and a longer half-life time (30–40 μs) [19]. PMS with an asymmetric structure was usually water soluble and more oxidative [20]. PMS could be activated by UV, graphene oxide, transition metals, nanoscale magnetic, heat, and gamma radiation [21]. How to select an appropriate catalyst to efficiently activate the persulfates salt to generate reactive oxide species (ROS), correspondingly increase the degradation performance of organic pollutants was considered a crucial issue.

Metal-organic frames (MOFs) have been widely used in the activation of PMS to remove organic pollutants [22]. Considering its large pore sizes and high specific areas [23], ample active centers, and plentiful derivatives [24,25], MOFs have garnered increasing interest in the catalytic field. Many scholars have prepared the MOFs materials, e.g., a core-heteroshell structural magnetic composite of ZIF-67/Vanadium-titanium magnetite (VTM) was synthesized through a solvothermal method, efficiently activating PMS for the removal of levofloxacin (LVF) (removal rate reaching 93.3%) [7]. The Co-Fe/NC@GCS was prepared by crosslinking the glutaraldehyde crosslinked chitosan (GCS) with Co-Fe nitrogen-doped carbon (derived from pyrolysis of MOF), efficiently removing 94.2% of sulfamethoxazole (SMX) within 60 min in the PMS solution [26]. The bone char (BBC) obtained through the pyrolysis of de-fatted swine bone could efficiently activate PS to the removal of 2,4-dichlorophenol (2,4-DCP) (removal rate reaching 85% within 30 min) [27]. The Fe-embedded biochar catalysts (Fe@HCBCs) originating from waste coffee grounds were used in the Fenton oxidation process for the removal of amaranth (AM) and sunset yellow (SY), with removal rates of 90.5% and 86.5%, respectively [28], to activate the persulfates to treat the organic pollutant-containing solution. However, the common problems, e.g., the weak dispersion and the poor separation and recycling properties of the catalysts hinder their actual application in the removal of organic pollutants [29,30]. As a result, some scholars combined the MOFs material with metal particles [31,32] and metallic oxide [33,34,35,36,37,38,39], aiming to improve the MOF’s actual application in wastewater treatment. However, the previously used metal or metallic oxide particles would usually need to be resynthesized, with more costs.

Herein, aiming to synchronously achieve the goal of the utilization of natural waste and activation of PMS, we have directly combined the MOFs material with waste coffee grounds (CG), analyzing the potential of CG in the forming of the MOF@CG composite. A series of material characterization methods including X-ray diffraction (XRD), X-ray photoelectron spectroscopy (XPS), Fourier transform infrared (FTIR), and Scanning electron microscopy (SEM) were conducted to analyze the structural performance of the prepared CG@ZIF-67. The primary reactive oxide species (ROS) in the degradation system were identified using electron paramagnetic resonance (EPR) analysis and free radical quenching experiments.

## 2. Materials and Methods

### 2.1. Raw Materials and Chemical Reagents

Tetracycline Hydrochloride (TC, C_18_H_20_FN_3_O_4_, 98%) and PMS (KHSO_5_⋅0.5KHSO_4_⋅0.5K_2_SO_4_) were applied by Aladdin Biochemical Co., Ltd., Shanghai, China. The coffee grounds (CG) were directly collected from a certain coffee shop in the city of Zhengzhou, Henan province, China. The collected raw CG material was firstly crushed through ball mill crushers and then washed using the deionized water several times, until the impurities (the residual oil and surface dust) were totally eliminated. Then, the coffee grounds were dried under 80 °C in the drying oven until the constant weight was reached. Finally, the dried coffee grounds were sieved through a 100-mesh sieve and stored for the following experiment.

The reagents of 2-Methylimidazole (2-MeIm, C_4_H_6_N_2_, 98%), Co(NO_3_)_2_·6H_2_O (98.5%) were purchased from Sinopharm Chemical Reagent Co., Ltd., Shanghai, China. The reagents of methyl alcohol (CH_3_OH), NaNO_3_, KH_2_PO_4_, tert-butyl alcohol (TBA, C_4_H_10_O), and furfuryl alcohol (FFA, C_5_H_6_O_2_) were also supplied by Sinopharm Chemical Reagent Co., Ltd., Shanghai, China, with analytical grades. The sodium chloride (NaCl, 99.5%), sodium hydroxide (NaOH, 96%), ethanol absolute (EtOH, C_2_H_6_O, 99.7%), sodium sulfate (Na_2_SO_4_, 99%), sodium bi-carbonate (NaHCO_3_, 99.5%), and sulfuric acid (H_2_SO_4_, 98%) were bought from Tianjin Kemiou Chemical Reagent Co., Ltd., Tianjin City, China. The deionized water used in this study was supplied by Wahaha Group Co., Ltd., Hangzhou, China. All the above reagents were directly supplied by the chemical reagent company, without further purification.

### 2.2. Synthesis of CG@ZIF-67 Composite

**Figure 1:**
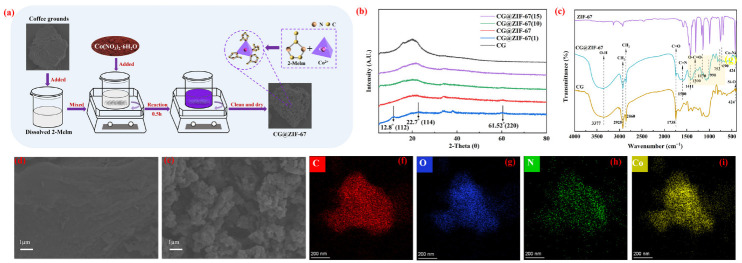
(**a**) schematic diagram for the preparation of the CG@ZIF-67 composite. (**b**) XRD spectra of the CG and prepared CG@ZIF-67. (**c**) FTIR spectra of ZIF-67, CG and prepared CG@ZIF-67. The SEM image of original CG (**d**) and prepared CG@ZIF-67 (**e**). EDS-mapping of CG@ZIF-67 (**f**–**i**) (Symbols of f, g, h, and i were presenting the element of C, O, N, and Co, respectively.

The proposed flowsheet of preparation of the CG@ZIF-67 composite was briefly depicted in Figure 1a. The CG@ZIF-67 composite was prepared through a facile solvothermal method [40]. The 2-MeIm (0.328 g) was firstly dissolved into 50 mL of the deionized water in a beaker (50 mL). Then, the CG (150 mg) was quickly added into the 2-MeIm solution and stirred in the thermostatic heating magnetic stirrer at 250 rpm for 5 min at 30 °C. Afterwards, the Co(NO_3_)_2_⋅6H_2_O (0.194 g) was directly added into the slurry containing CG and 2-MeIm solution and continuously stirred in the thermostatic heating magnetic stirrer at 250 rpm for 30 min. At the terminal of the reaction, the prepared CG@ZIF-67(5) composite was separated by centrifugation and then washed using methanol under ultrasound for a certain time, until the remaining supernatant was colorless. The prepared CG@ZIF-67(5) composite was finally dried in the vacuum drying oven at 60 °C for 12 h. For the change from Co(NO_3_)_2_⋅6H_2_O to CG samples as 5, 10, and 15, the prepared CG@ZIF-67 samples were labeled as CG@ZIF-67(1), CG@ZIF-67(10), and CG@ZIF-67(15), respectively.

### 2.3. TC Degradation Batch Experiment

During the TC degradation batch experiment, a certain amount of CG@ZIF-67 was added into the TC solution (20 mg/L, 100 mL) in a beaker (250 mL) and stirring at 300 rpm in the water bath thermostatic shaker at an indoor temperature (20 °C) to ensure the complete mixing between the CG@ZIF-67 and TC in the solution. Then, setting amounts of PMS solutions were added into the slurry (containing the TC and CG@ZIF-67) to start the TC degradation experiments. At the setting time intervals of 0, 1, 2, 3, 4, 5, 10, 15, 20, and 30 min, the slurry samples were immediately withdrawn from the reaction mixture. The withdrawn samples were quickly passed through a 0.45 μm syringe filter and immediately measured using the Ultraviolet-visible spectrophotometer (UV1800PC, Shanghai, China) at a wavelength of 357 nm (Figure S8). All the individual TC degradation experiments were repeated three times. The concentration of Co ions in the residual solution after TC degradation was measured using an inductively coupled plasma-atomic emission spectroscopy (ICP-OES, Optima 7000, PerkinElmer, Wellesley, MA, USA). The remaining PMS concentration in the solution was determined using the simple iodometric method by using KI and NaHCO_3_ as the reducing agents [41]. The main ROS (reactive oxygen species) acting in TC degradation were measured using electron paramagnetic resonance (EPR). The 5,5-dimethyl-1-pyrroline-N-oxide (DMPO) was used to quench the ·SO_4_^−^ and ·OH, and the 2,2,6,6-tetramethyl-4-piperidine (TEMP) would quench the ^1^O_2_. The EPR spectra were obtained by using the JES-X320 EPR spectrometer (microwave frequency, 9.83 GHz; microwave power, 2.00 mW, JES-X320, JEOL, Tokyo, JPN).

#### 2.3.1. Effect of CG@ZIF-67 Dosage on TC Degradation

The effects of the CG@ZIF-67 dosage on TC degradation were conducted through six samples. The six samples were conducted by adding a setting dosage of CG@ZIF-67 composite (0, 20, 60, 80, 100, and 120 mg/L) into the slurry containing TC (20 mg/L, 100 mL) in the 250 mL beakers. Then, the PMS (1.0 mM) solution was added into the slurry to start the TC degradation process. The contact temperature and initial pH were 293.15 K and 6.2.

#### 2.3.2. Effect of PMS Dosage on TC Degradation

The effect of the PMS dosage on the TC removal performance were conducted by adding a setting dosage of PMS (0, 0.2, 0.6, 0.8, 1.0, and 1.2 mM) into the slurry containing CG@ZIF-67 composite (100 mg/L) and TC (20 mg/L, 100 mL) in the 250 mL beakers to start the TC degradation process. The contact temperature and initial pH were 293.15 K and 6.2, respectively.

#### 2.3.3. Effect of Temperature and Initial pH on TC Degradation

The effect of temperature on the TC removal performance were investigated using four samples. The four samples were conducted by setting a series of temperatures (293.15, 303.15, 313.15, and 323.15 K). The factors including PMS concentration, initial TC concentration, and CG@ZIF-67 dosage were controlled as 1.0 mM, 20 mg/L, and 100 mg/L.

The effect of the initial pH on the TC removal performance were conducted using seven samples. The seven samples were controlled by setting a series of initial pH (3, 5, 6.2, 7, 9, 10, and 11) by adjusting H_2_SO_4_ (0.1 mM) and NaOH (0.1 mM) before the addition of PMS. Other conditions including the PMS concentration, CG@ZIF-67 dosage, and TC concentration were 1.0 mM, 100 mg/L, and 20 mg/L, respectively. The contact temperature was controlled as 293.15 K.

#### 2.3.4. Effect of Co-Existing Ions on TC Degradation

The effects of the natural coexisting ions including Cl^−^, HCO_3_^−^, NO_3_^−^, SO_4_^2−^, and H_2_PO_4_^−^ on the TC degradation were investigated by separately adding coexisting ion solutions (1 mM, 5 mM, 10 mM, and 20 mM) into the TC/CG@ZIF-67/PMS system at an unadjusted pH 6.2. The contact temperature and time were determined as 293.15 K and 30 min, respectively.

#### 2.3.5. Reusability of CG@ZIF-67 Composite

The reusability tests for the prepared CG@ZIF-67 were conducted for three cycles (Figure S4). The CG@ZIF-67 composite samples after every cycle of TC degradation were directly separated from the residual slurry and washed three times using the deionized water. The separated CG@ZIF-67 composite was then added into the TC solution to start the next cycle of TC degradation. Other conditions including the PMS dosage, contact temperature, and initial pH were kept as the first cycle.

### 2.4. Material Characterization of CG@ZIF-67 Composite

The X-ray diffraction (XRD) patterns of the prepared CG@ZIF-67 composite were obtained by using D/max 2500pc (Rigaku, Empyrean, The Netherlands) at 2θ ranging from 5 to 80°. The X-ray photoelectron spectroscopy (XPS, Escalab 250Xi, Thermo Scientific, Waltham, MA, USA) was used to analyze the chemical state of the main elementals of the prepared CG@ZIF-67. The morphology of the prepared CG@ZIF-67 was obtained by using a scanning electron microscope (SEM, Merlin compact, Zeiss, Jena, Germany). The total pore volume and pore size distribution of the CG@ZIF-67 composite were obtained through the N_2_ adsorption/desorption process (−196 °C) on the ASAP (2460, Micromeritics Instrument Ltd., Norcross, GA, USA).

## 3. Results

### 3.1. The Role of CG in the Prepared CG@ZIF-67 Composite

As shown in Figure 1b, the main characteristic peaks of the CG and CG@ZIF-67 composite were similar, confirming that CG was acting as the main component in the prepared CG@ZIF-67 composite. The peaks for CG@ZIF-67(1) located at 2θ of 12.8°and 22.7° were mainly related to the sodalite structure of ZIF-67 (pdf#43–0144). The peak observed 61.52° was mainly corresponding to the CoO (JCPDS, NO. 43-1004) [30]. However, at varying ratios of ZIF-67 to CG, the prepared CG@ZIF-67 was presenting a certain amorphous state, confirming that the stability of the prepared CG@ZIF-67 composite was needed to be improved. The FTIR spectra for CG and prepared CG@ZIF-67 composite were presented in Figure 1c. The bands observed at 1588 cm^−1^ and 752 cm^−1^ were related to the stretching of C=N and the vibration of the imidazole ring of 2-Melm (ZIF-67), respectively [42,43]. The vibrations observed at 600–1500 cm^−1^ were related to the stretching of the entire imidazole ring [44]. The band observed at 424 cm^−1^ was related to the stretching vibration of Co-N, attributing to the existence of ZIF-67 formed from the combination of the Co(NO_3_)_2_·6H_2_O and the 2-Melm [45]. The bands observed at 3377 cm^−1^, 1738 cm^−1^, and 1300 cm^−1^ were ascribed to the stretching vibration of O–H band, C=O band, and O-C=O band of CG, respectively [46]. All the characteristic peaks of ZIF-67 and CG were preserved for the CG@ZIF-67 composite, suggesting that the ZIF-67 was successfully loaded on the surface of CG. The SEM images of the original CG and prepared CG@ZIF-67 composite were shown in Figure 1d,e. Compared with the original CG, the layers of the ZIF-67 particles were grown on the surface of CG. The EDS-mapping images (Figure 1f–i) of the CG@ZIF-67 confirmed the distribution of C, N, and Co that the elements in the load layer (ZIF-67) were mainly C, N, O, and Co in the prepared CG@ZIF-67 composite. The specific surface areas of original CG and CG@ZIF-67 were determined as 0.11 and 4.02 m^2^ g^-1^ (Figure S1), respectively.

**Figure 2:**
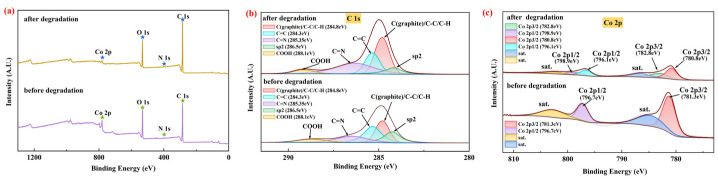
XPS spectra of the CG@ZIF-67 composite: (**a**) full-range spectra, (**b**) C 1s spectra, (**c**) Co 2p spectra.

The XPS spectra of the prepared CG@ZIF-67 composite were shown in Figure 2a–c. The full survey XPS spectra of CG@ZIF-67 (Figure 2a) confirmed the main element state of C 1s, N 1s, O 1s, and Co 2p in the prepared CG@ZIF-67 composite. As shown in the C 1s spectra (Figure 2b), the peaks observed at 284.3, 284.8, 285.35, 286.5, and 288.1 eV were mainly related to sp2, C(graphite)/C-C/C-H, C=C, C=N, and COOH [27,47,48,49], respectively, further confirming of the presence of C=N (in ZIF-67) in the prepared CG@ZIF-67 composite. In the Co 2p spectra (Figure 2c), the two main peaks at 781.3 and 796.7 eV corresponded to the Co 2p3/2 and Co 2p1/2, respectively, with two satellite peaks of 2p3/2 observed at 786.5 eV and 802.8 eV (Sat.) [50,51]. The N 1s peak at 397.6 eV was corresponding to N of 2-Melm ligand coordinated with Co (Figure S2). The XPS spectra result illustrated that the Co was mainly presented as Co(II) in the prepared CG@ZIF-67. These above results confirmed that the ZIF-67 had part ly grown on the surface of the CG. 

### 3.2. Catalytic Performance of CG@ZIF-67

**Figure 3:**
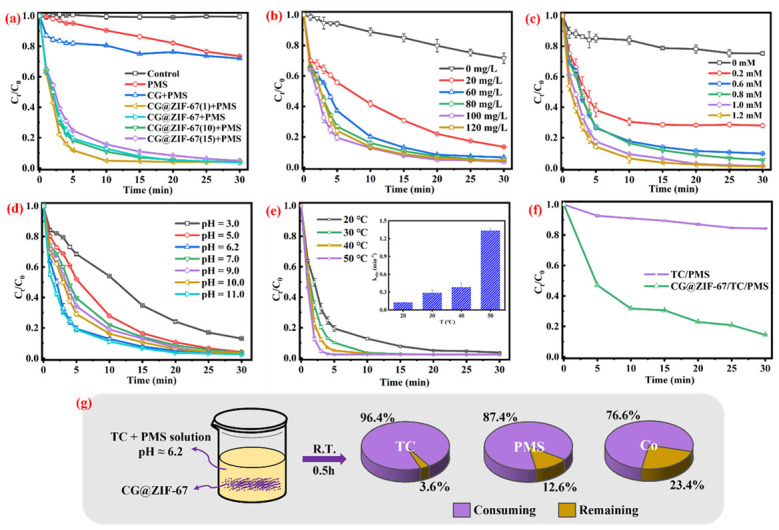
(**a**) TC removal efficiency in different systems, (**b**) CG@ZIF-67 dosage, (**c**) PMS dosage, (**d**) effect of initial pH, and (**e**) contact temperature on TC removal efficiency (inserter figure was K_obs_ versus temperature); (**f**) the consumption of PMS of different systems within 30 min; (**g**) the consuming and remaining content of TC, PMS, and the Co ions within 30 min in the CG@ZIF-67/PMS/TC system. Other conditions: [TC] = 20 mg/L and unadjusted pH 6.2.

The TC removal efficiency in different degradation systems were conducted and shown in Figure 3a. In the single PMS solution, 26.5% of the TC was removed, confirming that the single PMS could partially consume the TC in the aqueous solution. In the CG/PMS system, 27.9% of the TC was removed, indicating that the single CG could not efficiently active the PMS to realize the TC degradation in the solution. In the Co^2+^/PMS system, 71.4% of TC was removed (Figure S5). The TC removal ratio was determined as 96.3% in the CG@ZIF-67/PMS system within 30 min, confirming the complete consumption of TC in the CG@ZIF-67/PMS system. As shown in Figure 2f, the consumption ratio of PMS in the CG@ZIF-67/PMS system could reach 85.6% and 11.1% of TOC was removed (Figure S7), while only 15.8% of the PMS was consumed in the PMS/TC system, indicating that the CG@ZIF-67 catalyst could efficiently active PMS to degrade TC in an aqueous solution. The TC removal efficiencies of CG@ZIF-67(1), CG@ZIF-67(10), and CG@ZIF-67(15) in the PMS solution were determined as 96.1%, 95.9%, and 95.0%, thus indicating that different ratios of ZIF-67 in the prepared CG@ZIF-67 would not significantly change the TC removal rate.

#### 3.2.1. Effect of CG@ZIF-67 Composite and PMS Dosage

With the CG@ZIF-67 dosage ranging from 20 mg/L to 60 mg/L in the PMS solution (1.0 mM) (Figure 3b), the TC removal efficiency was increased from 86.5% to 95.9%, which was attributed to the catalytic active sites of PMS activation increasing the CG@ZIF-67 dosage. However, with the CG@ZIF-67 dosage further increasing to more than 60 mg/L, the TC degradation ratio was nearly kept constant. This result was mainly attributed to it, indicating that more catalyst would activate PMS to generate more ·SO_4_^−^, while the excess catalyst would undergo a self-quenching process or consume the remaining PMS, as shown in Equations (1) and (2) [52,53]. With the PMS dosage ranging from 0 to 1.2 mM (Figure 3c), the TC removal efficiency was increased from 24.8% to 98.9%. With the PMS dosage further increasing to more than 1.0 mM, the TC degradation efficiency remained nearly constant, which was attributed to the insufficient dosage of the CG@ZIF-67 catalyst.

SO_4_^2−^ + ·SO_4_^−^ → S_2_O_8_^2−^, k = 5 × 10^8^ M^−1^s^−1^ (1)

S_2_O_8_^2−^+ ·SO_4_^−^ → ·S_2_O_8_^−^+ SO_4_^2−^, k = 5.5 × 10^5^ M^−1^s^−1^ (2)

#### 3.2.2. Effect of Initial pH

The effect of pH on the TC removal efficiency was shown in Figure 3d. Under the natural pH (pH of 6.2), approximately 96.3% of the TC was removed in the CG@ZIF-67/PMS system within 30 min. At the more acidic pH of 3.0, the TC removal efficiency was decreased to 87.0%, which was attributed to the quenching of ·OH and ·SO_4_^−^ by the excessive H^+^ (Equations (3) and (4)) [54,55,56], accordance with the higher leaching rate of Co (at pH of 3) (Figure S6). At pH of 5.0, 7.0, 9.0, 10.0, and 11.0, the TC removal efficiency was all kept at a relatively higher value (more than 95.0%), presenting a high tolerance to pH in an aqueous solution because the PMS could be efficiently activated under strongly basic conditions. At a pH of 11.0 (>9.4), the PMS was decomposed into the strong oxidant of SO_5_^2−^. The generated SO_5_^2−^ was easier to be activated by a catalyst, compared with PMS (HSO_5_^−^), further increasing the TC removal efficiency [57,58,59]. Furthermore, the non-free radical of ^1^O_2_ was dominant in the CG@ZIF-67/PMS system, which was weakly dependent on the pH of the solution [60].

H^+^ + ·OH + e^−^ → H_2_O, k = 7 × 10^9^ M^−1^s^−1^ (3)

·SO_4_^−^ + H^+^ + e^−^ →·HSO_4_^−^ (4)

#### 3.2.3. Effect of Contact Temperature

The TC degradation process was accelerated with the increasing reaction temperature (Figure 3e), while the k_obs_ of TC degradation gradually increased with the increasing temperature (Table S2). The higher temperature would provide more energy to overcome the energy barrier of PMS activation, promoting the generation of free radicals [28].

The activation energies (Ea) of TC degradation in PMS activated by CG@ZIF-67 were evaluated by plotting ln (k_obs_) against 1/T (Figure S3, Equation S2). The calculated Ea (56.57 kJ/mol) was higher than that of the diffusion controlled reaction (ranging within 10–13 kJ/mol) [61], implying the TC removal was mainly controlled by the chemical reaction, rather than the mass transfer process.

As shown in Figure 3g, the TC removal efficiency was 96.3%, while 87.4% of the PMS and 76.6% of the Co ions were consumed within 30 min in the CG@ZIF-67/PMS/TC system.

### 3.3. Effect of Co-Existing Substances

**Figure 4:**
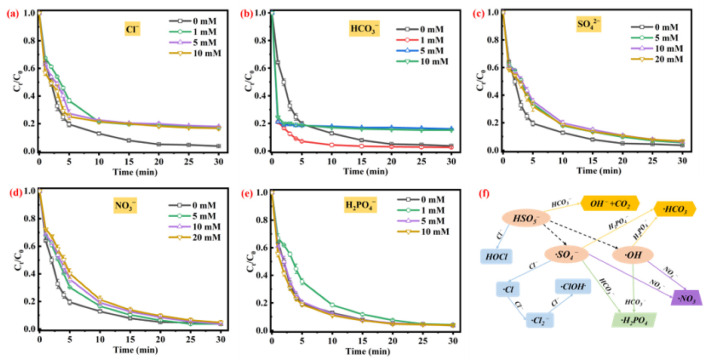
Effects of co-existing substances (commonly found in natural environment) on TC removal efficiency: (**a**) Cl^−^, (**b**) HCO_3_^−^, (**c**) SO_4_^2−^, (**d**) NO_3_^−^, and (**e**) H_2_PO_4_^−^. Conditions: [PMS] = 1.0 mM, [catalyst] = 100 mg/L, [TC] =20 mg/L, unadjusted pH 6.2, and contact temperature of 293.15 K. (**f**) The proposed mechanisms for inorganic ions affecting the active species generation in AOPs.

The effects of anions (Cl^−^, HCO_3_^−^, NO_3_^−^, SO_4_^2−^, and H_2_PO_4_^−^) on the TC removal efficiency was shown in Figure 4. As shown as in Figure 4a, compared with the control group (TC removal efficiency of 96.3%), the TC removal efficiency was sharply decreased to 82.9% with the addition of Cl^−^ (1 mM) to the CG@ZIF-67/PMS system, which was mainly attributing to the elimination of ·OH and ·SO_4_^−^ by the added Cl^−^; this was calculated using Equations (5)–(8) [62,63,64,65]. Furthermore, the added Cl^−^ could also consume the PMS, according to Equations (9) and (10) [66,67], thus decreasing the TC degradation efficiency.

Cl^−^ + ·OH → ·ClOH^−^, k = 4.3 × 10^9^ M^−1^s^−1^ (5)

·ClOH^−^ → Cl^−^+ ·OH, k = 6.1 × 10^9^ M^−1^s^−1^ (6)

Cl^−^ + ·SO_4_^−^ → SO_4_^2−^ + ·Cl (7)

·Cl + Cl^−^ → ·Cl_2_^−^, k=8 × 10^9^ M^−1^s^−1^ (8)

Cl^−^ + HSO_5_^−^ → SO_4_^2−^ + HOCl (9)

2Cl^−^ + HSO_5_^−^ + H^+^ → SO_4_^2−^ + Cl_2_ + H_2_O (10)

HCO_3_^−^ + HSO_5_^−^ → ·SO_4_^−^ + 2OH^−^ + CO_2_ (11)

HCO_3_^−^ + HSO_5_^−^ → HSO_4_^−^ + HCO_4_^−^ (12)

Compared with the control experiment, the TC removal ratio with the addition of 1 mM HCO_3_^−^ was slightly increased to 97.6%, due to the generation of free radicals by the activation of HCO_3_^−^ (Equation (11)) [68,69]. In addition, the HCO_3_^−^ could react with PMS to form percarbonates (Equation (12)), which could selectively degrade organic pollutants [70,71]. An increased concentration of HCO_3_^−^ (5 mM and 10 mM) significantly decreases the TC removal efficiency in the PMS/CG@ZIF-67 system (Figure 4b), which is mainly attributed to the consumption of ·OH and ·SO_4_^−^ by the added HCO_3_^−^ [72]. Other anions including NO_3_^−^, SO_4_^2−^, and H_2_PO_4_^−^ (Figure 4c–e) presented a weak effect on the TC removal process in the CG@ZIF-67/PMS. The NO_3_^−^ could partially consume the free radicals [53]. The addition of SO_4_^2−^ would quench part of the ·SO_4_^−^, while the ·SO_4_^−^ would be converted to S_2_O_8_^2−^ [73]. Furthermore, part of the ·OH would also be consumed by SO_4_^2−^ [74]. The H_2_PO_4_^−^ could also consume part of the free radicals (·OH and ·SO_4_^−^) [69,75]. Furthermore, the H_2_PO_4_^−^ could complex with Co(II), reducing the catalytic activity of CG@ZIF-67 [76].

### 3.4. Identification of Reactive Specie

**Figure 5:**
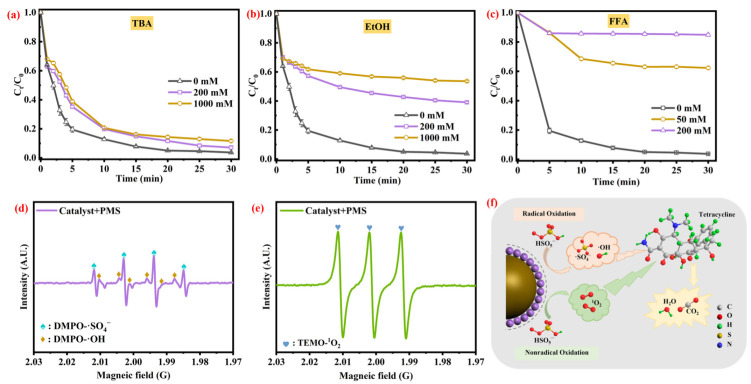
Effects of different radical scavengers on the TC degradation in CG@ZIF-67/PMS system (**a**) TBA, (**b**) EtOH, and (**c**) FFA. EPR spectra of ·OH, ·SO_4_^−^ (**d**), and ^1^O_2_ (**e**) in CG@ZIF-67/PMS/TC system. Conditions: [PMS] = 1.0 mM, [catalyst] = 100 mg/L, [TC] = 20 mg/L, unadjusted pH 6.2, and contact temperature of 293.15 K. (**f**) The proposed mechanism diagram for active species consuming the TC.

As shown in Figure 5a–c, with the addition of TBA (200 mM and 1000 mM), the TC removal efficiency has a slight drop (92.9% and 88.9%), respectively, which indicates that only a small amount of ·OH was generated in the CG@ZIF-67/PMS/TC system. With EtOH (200 mM and 1000 mM) added to the system, the TC removal efficiency was decreased to 61.1% and 46.6%, respectively. This result indicated the ·SO_4_^−^ would facilitate the TC removal process. With addition of FFA (50 mL and 200 mM), the TC removal efficiency was sharply decreased from 37.7% and 14.6%, respectively, confirming the crucial role of ^1^O_2_ in the TC degradation process.

The EPR spectra conducted by using DMPO and TEMP as the spin-trapping agent were shown in Figure 5d,e, while the signals of DMPO-·OH and DMPO-·SO_4_^−^ were both observed in the CG@ZIF-67/PMS system. The characteristic signals of DMPO-·OH (αN = αH = 14.9 G) confirmed the minor presence of ·OH in the TC degradation process [77]. The spectra of DMPO-·SO_4_^−^ (αH = 9.6 G, αH = 1.48 G, αH = 0.78 G, and αN = 13.2 G) further confirmed the presence of ·SO_4_^−^ in the CG@ZIF-67/PMS system [78]. The characteristic peaks of ^1^O_2_ EPR spectra (the characteristic peaks of ^1^O_2_ αN = 16.9 G) were clearly observed, further confirming the key role of ^1^O_2_ in the TC degradation process [79,80], which is in accordance with the quenching experiments result. Through those above analyses, the ^1^O_2_ would play a dominant role in the TC degradation process, with a non-radical activation route. Compared with the non-radical activation route, the ·SO_4_^−^ would be a relatively weak driver of the TC degradation process; the reactive species contributing the most were ^1^O_2_ and ·SO_4_^−^.

### 3.5. Insight into the PMS Activation by CG@ZIF-67 Composite

The Co^2+^ ions (on the surface of CG@ZIF-67 composite) could directly activate PMS to form the ROS of ·OH and ·SO_4_^−^. In addition, from the XPS results (Figure 2c), the Co^2+^ captured water molecules to generate Co–OH and the CoOH^+^ of the CG@ZIF-67 composite would facilitate the redox cycle of PMS in the CG@ZIF-67/PMS/TC system, thus accelerating the activation of HSO_5_^−^ to form ·SO_4_^−^, which was calculated using the following Equations (13)–(15) [81]. The ^1^O_2_ was firstly originated from the self-decomposition of PMS on the surface of CG@ZIF-67, where the generated ·OH and ·SO_4_^−^ could decompose HSO_5_^−^ to ^1^O_2_ as shown in Equations (16)–(18) [77,82]. Furthermore, ·SO_5_^−^ would oxidize the HSO_5_^−^ and H_2_O to form ^1^O_2_, which can be described by Equations (18) and (19) [83,84,85,86].

≡Co(II) + H_2_O → CoOH^+^ + H^+^ (13)

CoOH^+^ + HSO_5_^−^ → ≡CoO^+^ + ·SO_4_^−^ + H_2_O (14)

CoO^+^ + 2H^+^ → ≡Co^3+^ + H_2_O (15)

2·OH + HSO_5_^−^ → HSO_4_^−^ + ^1^O_2_ + H_2_O (16)

·SO_4_^−^ + HSO_5_^−^ → SO_4_^2−^ + ·SO_5_^−^ + H^+^, k < 1 × 10^5^ M^−1^s^−1^ (17)

HSO_5_^−^ + SO_5_^2−^ → 2SO_4_^2−^ + ^1^O_2_ + H^+^, k = 0.2 M^−1^s^−1^ (18)

2·SO_5_^−^ + H_2_O → 2H^+^ + 2SO_4_^2−^ + 1.5^1^O_2_ (19)

We should note that, from the XPS results (Figure 2b), the -COOH content of the CG@ZIF-67 decreased from 15.1% to 11.2% and the C(graphite)/C-C/C-H increased from 22.7% to 29.3% within the TC removal process (Table S1), indicating that the O-C=O bond could decompose the PMS to form ·SO_4_^−^ [48,87,88]. The content of the graphite N was decreased from 36.9% to 23.2%, while the C sp2 decreased from 20.5% to 13.3% within the TC removal process. Furthermore, the content of pyridinc N was enhanced from 47.8% to 60.4% (Table S1). The content change confirmed the participation of N (derived from CG) in driving the TC removal process. The graphite N of the CG@ZIF-67 composite might facilitate the electron transfer from the C atom to the N atom, further breaking the chemical inertia of sp2 carbon. The positive carbon atoms tend to adsorb HSO_5_^−^ to form into ·OH and ·SO_4_^−^ [89,90,91,92,93].

## 4. Conclusions

In this paper, aiming to synchronously achieve the goals of natural coffee ground reuse and MOF material catalytic performance enhancement, the CG@MOF composites were successfully prepared and applied in the activation of PMS to TC degradation in an aqueous solution.

The material characterization routes including XRD, FTIR, XPS, and EDS-mapping confirmed the growth of ZIF-67 particles on the surface of the CG. The TC removal efficiency with CG@ZIF-67 composite acting in PMS activation could almost reach 96.3%, while the ^1^O_2_ and ·SO_4_^−^ would play the crucial role to TC removal, withstanding a relatively wider pH range (3–11).

Compared with advanced conventional material oxidation technology, the TC could be effectively degraded at a wide pH range, thus confirming that the CG@ZIF-67/PMS system could achieve a satisfactory catalytic performance over a wide range of pH values and further ensure the possibility of its practical applications.

## Data Availability

Not applicable.

## Supplementary Materials

The following supporting information can be downloaded at: https://www.mdpi.com/article/10.3390/toxics11020088/s1, Figure S1: N_2_ adsorption–desorption isotherms of the original CG (a) and prepared CG@ZIF-67 composite (b). Figure S2: XPS spectra of the CG@ZIF-67 composite: (a) N 1s and (b) O 1s. Figure S3: Reaction activation energy of temperature on TC removal efficiency. Figure S4: Recyclability of CG@ZIF-67 (a) and the Co ions concentration in three batch experiments (b). Figure S5: The TC removal efficiency in the Co^2+^/PMS system. Figure S6: The correlation between Co leaching and the pH. Figure S7: TOC removal after 30 min reaction in CG@ZIF-67/PMS/TC system. Figure S8: The comparison for the display data between the HPLC and UV-Vis tests. Table S1: The element content of C, N, O, and Co (calculated using XPS analysis) in CG@ZIF-67 before and after TC removal process. Table S2: The K_obs_ of TC removal process in CG@ZIF-67/PMS system at different temperatures. Text S1. Determination of PMS concentration in the solution.
