# Supplementary material for: The Potential of Spent Coffee Grounds @ MOFs Composite Catalyst in Efficient Activation of PMS to Remove the Tetracycline Hydrochloride from an Aqueous Solution
Source: Toxics. 2023 Jan 17;11(2):88. doi: 10.3390/toxics11020088 (PMC9965720; doi:10.3390/toxics11020088)
Supplement: Supplementary file 1 [file toxics-11-00088-s001.zip › toxics-2127568-supplementary.pdf]

Supplementary Information

The potential of spent coffee grounds @ MOFs composite catalyst in efficient activation of PMS to remove the tetracycline hydrochloride from an aqueous solution

Wei Zhang [a,b,c,d,e,f,g], Jiajia Lu [a], Shoushu Liu [a], Chen Wang [a], Qiting Zuo [b,g], Lin Gong [a,c,d,e,f,g*]

[a] School of Ecology and Environment, Zhengzhou University, 100 Kexue Avenue, Zhengzhou 450001, P. R. China

[b] School of Water Conservancy Engineering, Zhengzhou University, Zhengzhou 450001, China

[c] Yellow River Institute for Ecological Protection and Regional Coordination Development, Zhengzhou University, 100 Kexue Avenue, Zhengzhou, Henan, 450001, PR China

[d] Henan Key Laboratory of Water Pollution Control and Rehabilitation Technology, Pingdingshan, Henan, 467036, PR China

[e] Henan International Joint Laboratory of Water Cycle Simulation and Environmental Protection, Zhengzhou, 450001, PR China

[f] Zhengzhou Key Laboratory of Water Resource and Environment, Zhengzhou, 450001, PR China

[g] Yellow River Institute for Ecological Protection and Regional Coordination Development, Zhengzhou University, 100 Kexue Avenue, Zhengzhou, Henan, 450001, PR China

* Corresponding Author:

Lin Gong, School of Ecology and Environment, Zhengzhou University, 100 Kexue Avenue, Zhengzhou, Henan, 450001, PR China

E-mail: lingo716@163.com

Number of pages: 8

Number of Tables: 2

Number of Figures: 8

Supplementary captions

Text S1. Determination of PMS concentration in the solution.

Table S1 The element content of C, N, O, and Co (calculated by XPS analysis) in CG@ZIF-67 before and after TC removal process.

Table S2. The K_{obs} of TC removal process in CG@ZIF-67/PMS system at different temperatures.

Figure S1. N_2 adsorption–desorption isotherms of the original CG (a) and prepared CG@ZIF-67 composite (b).

Figure S2. XPS spectra of the CG@ZIF-67 composite: (a) N 1s and (b) O 1s.

Figure S3. Reaction activation energy of temperature on TC removal efficiency.

Figure S4. Recyclability of CG@ZIF-67 (a) and the Co ion concentration in three batch experiments (b).

Figure S5. The TC removal efficiency in the Co^{2+}/PMS system.

Figure S6. The leaching concentration of Co with increasing pH.

Figure S7. TOC removal efficiency of CG@ZIF-67/PMS/TC system after 30 min.

Figure S8. The comparison for the display data between the HPLC and UV-Vis tests.

Text S1. Determination of PMS concentration in the solution.

The remaining PMS concentration in the solution after the TC removal process was determined by using the chemical reagents of KI and $NaHCO_3$ as reductants. The KI stock solution (10mM, 100 mL) was prepared by mixing 0.166 g KI and 0.04 g $NaHCO_3$ and diluting to 100 mL in a volumetric flask.

The solution after reaction (0.1 mL) was withdrawn, with the KI stock solution (4.9 mL) immediately added into the sample and mixed for 5 min; this was followed by analyzing using the Ultraviolet-visible spectrophotometer (UV1800PC, Shanghai, China) at a wavelength (λ_{max}) of 352 nm.

Table S1 The element content of C, N, O, and Co (calculated using XPS analysis) in CG@ZIF-67 before and after TC removal process.

Element		Before reaction	After reaction
C (at. %)	sp2	20.5	13.3
	C(graphite)/C-C/C-H	22.7	29.3
	C=C	22.6	20.5
	C=N	19.1	25.7
	COOH	15.1	11.2
	Total*	70.2	79.2
N (at. %)	Co-N	15.3	16.4
	pyridinic N	47.8	60.4
	graphitic N	36.9	23.2
	Total*	1.8	1.47
O (at. %)	C=O	60.6	72.6
	C–OH	39.4	27.4
	Total*	23.8	18.1
Co (at. %)	Co(II)	100	65.7
	Co(III)	0	34.3
	Total*	4.1	1.27

Table S2. The K_{obs} of TC removal process in CG@ZIF-67/PMS system at different temperatures.

Catalyst	T (°C)	k_{obs} (min^{-1})
CG@ZIF-67	20	0.1319
	30	0.3237
	40	0.4356

	50	1.3166

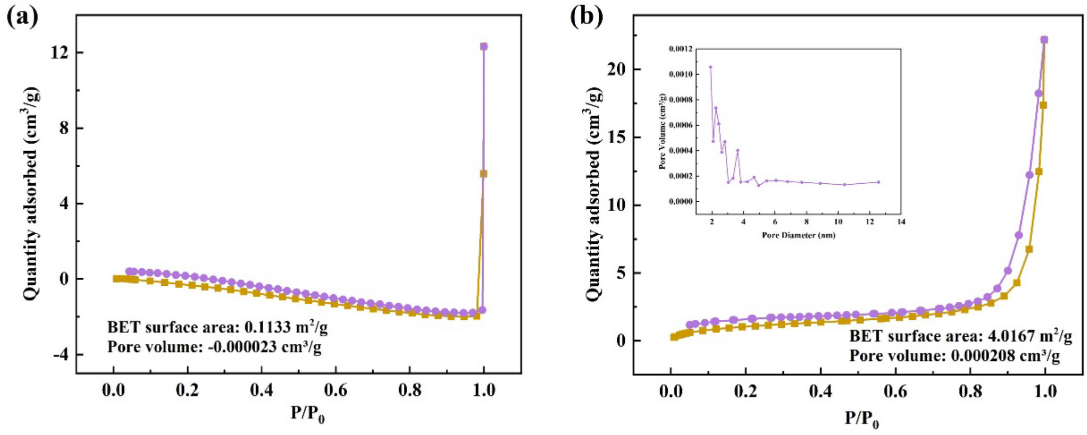

Figure S1. N_2 adsorption–desorption isotherms of the original CG (a) and prepared CG@ZIF-67 composite (b).

The surface area and pore-size distribution of the CG and CG@ZIF-67 composite were analyzed using the BET method and shown in Fig. S1. The specific surface areas of the original CG and CG@ZIF-67 were determined as 0.11 and 4.02 m^2 g^{-1}, respectively, indicating that the loading of the ZIF-67 particles on the surface of CG increased the specific surface of the prepared composite.

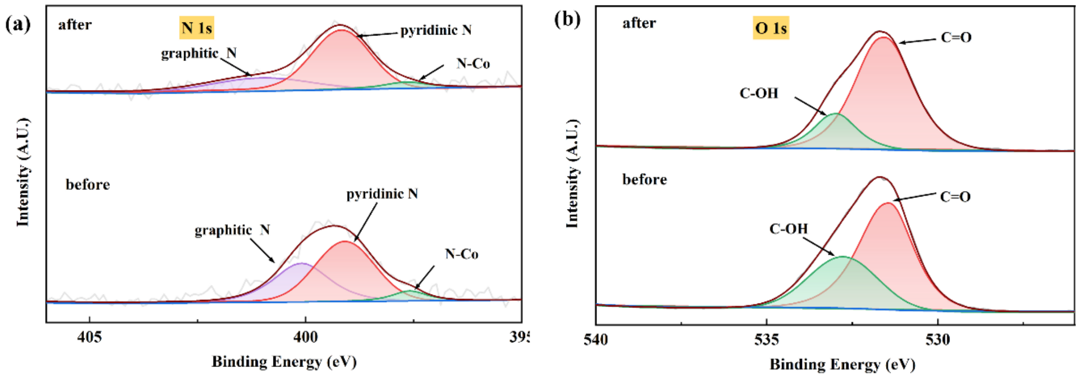

Figure S2. XPS spectra of the CG@ZIF-67 composite: (a) N 1s and (b) O 1s.

As shown in the N 1s spectra (Fig. S2a**)**, the N 1s peak at 397.6 eV was corresponding to N of 2-Melm ligand coordinated with Co. In the O 1s spectra (Fig. S2b), the two peaks observed at 531.4 and 533.6 eV were assigned to the lattice oxygen in C–OH (formed by a coordination reaction of exposed Co(II) and deprotonated H_2O

on the catalyst surfaces) and the Carbonyl double bond (C=O) [92], respectively.

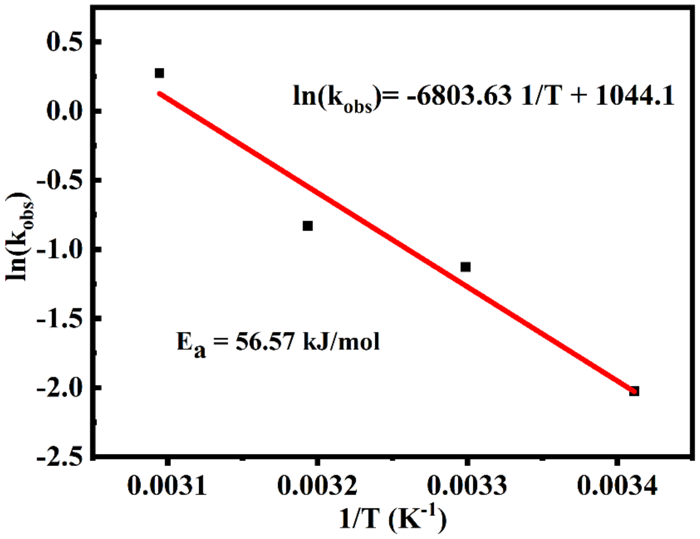

Figure S3. Reaction activation energy of temperature on TC removal efficiency.

The reaction rate constants (kobs) at different temperatures were calculated using the pseudo-first-order kinetics Equations (Equations S1), without standard errors in the calculations process:

$$\ln \frac{c_0}{c_t} = - k_{obs}\, t \qquad \textbf{(Eq. S1)}$$

$$\ln k_{obs} = \ln k_0 - \frac{E_a}{RT} \qquad \textbf{(Eq. S2)}$$

where c_0 and c_t is the initial and real-time concentration of organic pollution, E_a is the Arrhenius activity energy (KJ mol^{-1}), and the activation energies (Ea) of TC degradation in PMS activated by CG@ZIF-67 were evaluated by plotting ln (k_{obs}) against 1/T (Eq. S2, Fig. S3).

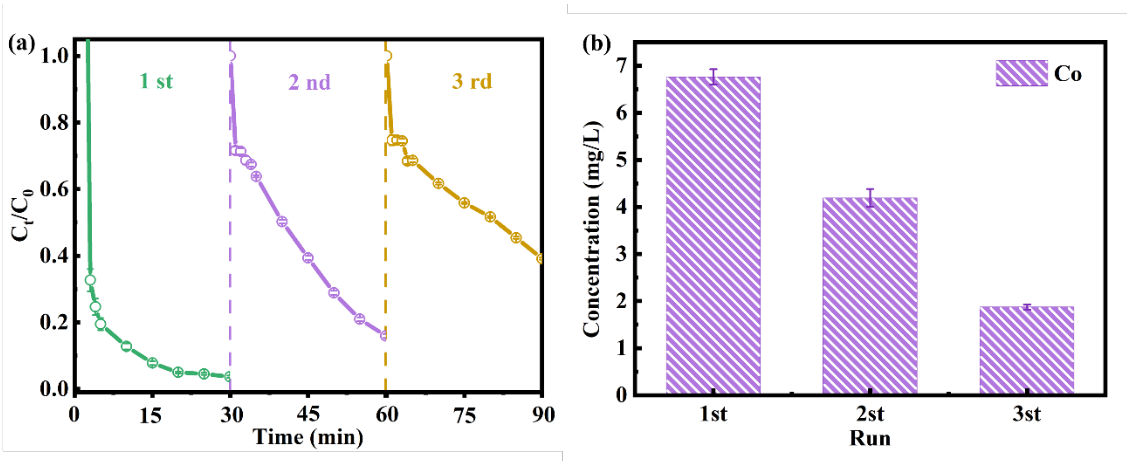

Figure S4. Recyclability of CG@ZIF-67 (a) and the Co ion concentrations in three batch experiments (b).

As shown in Fig. S4a, compared with the first cycle (TC removal efficiency of 96.3%), the TC removal efficiency decreased to 84.1% at the second cycle. At the third cycle, the TC removal efficiency decreased to 60.9% in the CG@ZIF-67/PMS system, indicating that CG@ZIF-67 showed an acceptable reusability performance. Under the first cycle, the concentration of Co ions in the residual solution after TC degradation was approximately 6.642 mg/L; with increasing cycle times, the concentrations of Co ions ranged from 4.055 mg/L to 1.834 mg/L (Fig. S4b), indicating that the stability of the prepared CG@ZIF-67 composite still needed to be further improved.

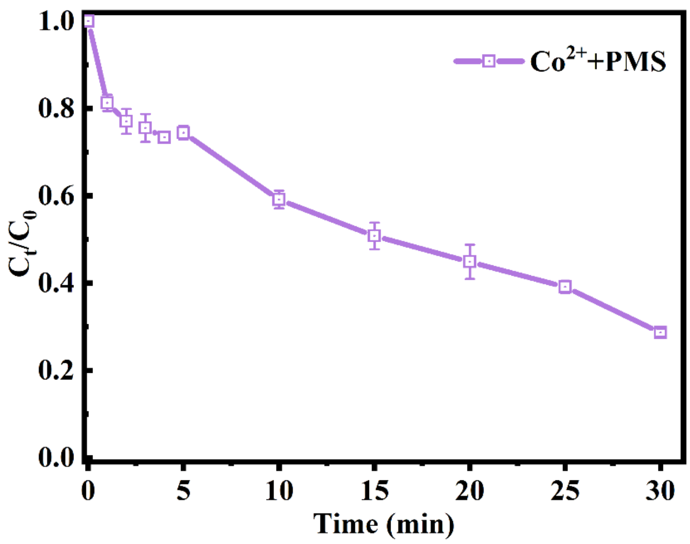

Figure S5. The TC removal efficiency in the Co^{2+}/PMS system. Conditions: [PMS] = 1.0 mM, [catalyst] = 100 mg/L, [TC] =20 mg/L, unadjusted pH 6.2, and contact temperature of 293.15 K.

As shown in Fig. S5, around 71.4% of the TC was removed in the Co^{2+}/PMS system, indicating that the single Co^{2+} could partly activate the PMS to realize the TC degradation in the solution.

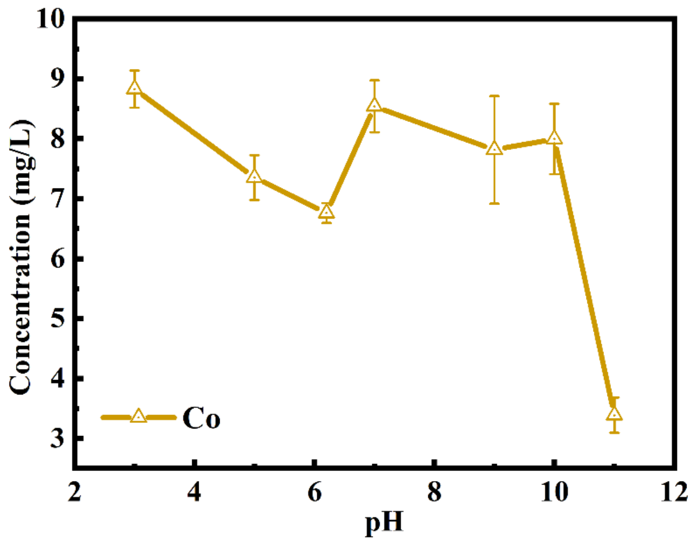

Figure S6. The leaching concentration of Co with increasing pH. Conditions: [PMS] = 1.0 mM, [catalyst] = 100 mg/L, [TC] =20 mg/L, and contact temperature of 293.15 K.

The leaching concentrations of Co with increasing pH were shown in Fig. S6. The leaching concentrations were relatively higher at pH less than 10, compared with pH of 11. At pH of 3.0, the TC removal efficiency was only 87.0%, which is in accordance with the higher leaching rate of Co (at pH of 3). However, at other pH conditions, the correlations of the TC removal efficiency and Co leaching ratio were quite limited.

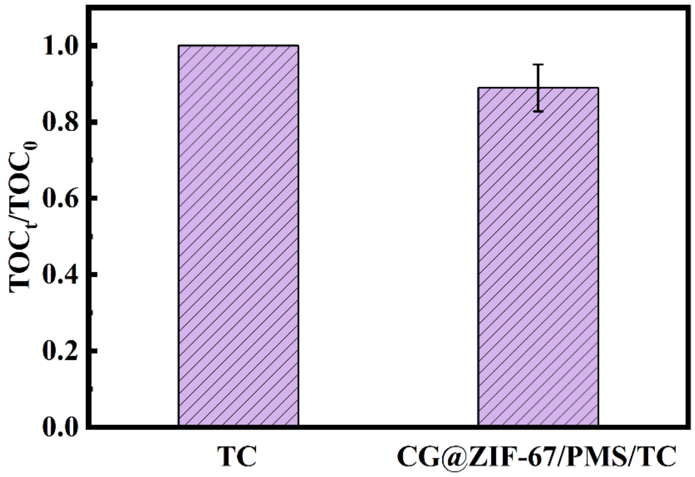

Figure S7. TOC removal efficiency of CG@ZIF-67/PMS/TC system after 30 min.

$$\text{Mineralization efficiency} = \frac{(TOC_0 - TOC_t)}{TOC_0} \times 100\% \qquad \textbf{(Eq. S3)}$$

where TOC_0 and TOC_t (mg/L) are the TOC value at the beginning and terminal of the

TC degradation process, respectively.

The TOC removal efficiency of the CG@ZIF-67/PMS/TC system was shown in Fig. S7; approximately 11.1% of the TOC was removed at a reaction time of 30 min in the CG@ZIF-67/PMS/TC system, confirming that future experiments are needed to enhance the TOC removal efficiency.

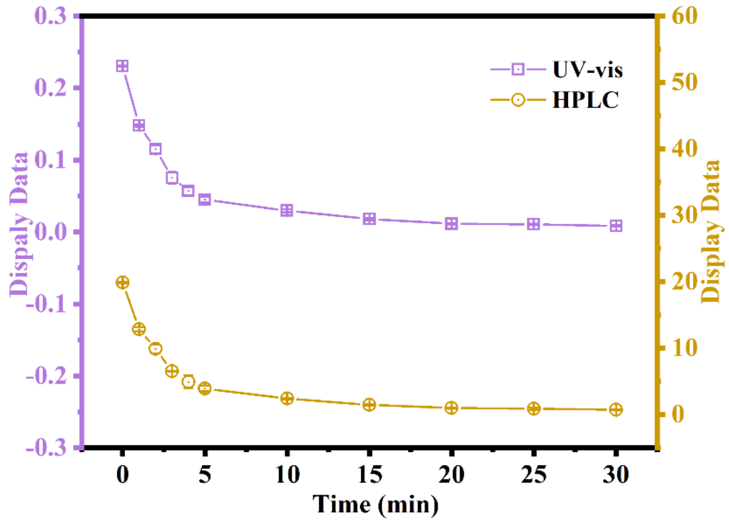

Figure S8. The comparison for the display data between the HPLC and UV-Vis tests. Conditions: [PMS] = 1.0 mM, [catalyst] = 100 mg/L, [TC] =20 mg/L, unadjusted pH 6.2, and contact temperature of 293.15 K.

The relevant literature has measured the concentration of TC by using the UV-vis spectrophotometry [13,16,25]. Furthermore, we have compared the correlations of HPLC and UV-vis data reading from our supplied experiment (Fig. S8). The comparison between the HPLC and UV-vis data reading indicated that the correlation for HPLC and UV-vis data versus the reaction time was similar. Thus, the concentration of TC was measured using UV-vis spectrophotometry in this experiment.
